# Three-dimensional Huh7 cell culture system for the study of Hepatitis C virus infection

**DOI:** 10.1186/1743-422X-6-103

**Published:** 2009-07-15

**Authors:** Bruno Sainz, Veronica TenCate, Susan L Uprichard

**Affiliations:** 1 Department of Medicine, The University of Illinois at Chicago, Chicago, IL 60612, USA; 2 Department of Microbiology and Immunology, The University of Illinois at Chicago, Chicago, IL 60612, USA

## Abstract

**Background:**

In order to elucidate how Hepatitis C Virus (HCV) interacts with polarized hepatocytes in vivo and how HCV-induced alterations in cellular function contribute to HCV-associated liver disease, a more physiologically relevant hepatocyte culture model is needed. As such, NASA-engineered three-dimensional (3-D) rotating wall vessel (RWV) bioreactors were used in effort to promote differentiation of HCV-permissive Huh7 hepatoma cells.

**Results:**

When cultured in the RWV, Huh7 cells became morphologically and transcriptionally distinct from more standard Huh7 two-dimensional (2-D) monolayers. Specifically, RWV-cultured Huh7 cells formed complex, multilayered 3-D aggregates in which Phase I and Phase II xenobiotic drug metabolism genes, as well as hepatocyte-specific transcripts (HNF4α, Albumin, TTR and α1AT), were upregulated compared to 2-D cultured Huh7 cells. Immunofluorescence analysis revealed that these HCV-permissive 3-D cultured Huh7 cells were more polarized than their 2D counterparts with the expression of HCV receptors, cell adhesion and tight junction markers (CD81, scavenger receptor class B member 1, claudin-1, occludin, ZO-1, β-Catenin and E-Cadherin) significantly increased and exhibiting apical, lateral and/or basolateral localization.

**Conclusion:**

These findings show that when cultured in 3-D, Huh7 cells acquire a more differentiated hepatocyte-like phenotype. Importantly, we show that these 3D cultures are highly permissive for HCV infection, thus providing an opportunity to study HCV entry and the effects of HCV infection on host cell function in a more physiologically relevant cell culture system.

## Background

Hepatitis C virus (HCV), a liver tropic positive-stranded RNA flavivirus, infects ~170 million people worldwide, causing acute and chronic hepatitis and hepatocellular carcinoma [1]. However, since its discovery in 1989, a major obstacle impeding HCV research has been the lack of robust cell culture and small animal infection models. Notably significant advancement has been made with the identification of a genotype 2a HCV consensus clone (Japanese Fulminant Hepatitis, JFH-1) that can replicate and produce infectious HCV in vitro in the Huh7 human hepatoma-derived cell line [2-4], allowing for the study of the entire viral life cycle. This system, however, is limited in that it makes use of a non-differentiated cell line that does not recapitulate the cellular conditions encountered by HCV in vivo [5,6]. In particular, hepatocyte polarity is likely relevant to HCV entry as growing evidence suggests interplay between HCV and tight junction (TJ) proteins claudin-1 (CLDN1) [7] and occludin [8,9] is essential for viral uptake. In fact, recent reports surprisingly suggests that hepatocyte polarity may restricts HCV entry [10,11]. While an inverse relationship between viral entry and hepatocyte polarity would potentially represent a unique determinant of HCV entry, to date attempts to dissect this relationship have been difficult and inconclusive due to the inability of cell culture grown hepatocyte-derived cell lines, such as Huh7 cells, to mimic the complex polarized phenotype of hepatocytes in vivo. To circumvent these restriction, studies investigating HCV entry into Caco-2 cells [10] and HepG2 cells [11] have been performed as these cells can polarize to differing degrees in vitro, however, neither Caco-2 or HepG2 cells supports efficient HCV infection limiting their utility. As such, a more physiologically relevant hepatocyte tissue culture model is still needed to assess if cell polarity negatively affects HCV infection.

The NASA-engineered RWV is a horizontally rotating cylindrical culture vessel which reduces shear and turbulence associated with conventional stirred bioreactors; therefore, it simulates aspects of microgravity similar to the environment encountered during fetal development [12-14]. In contrast to conventional static tissue culture systems, cells grown in the RWV are cultured in "suspended animation" where they are continuously free-falling [12,15]. Thus, while the 2-D environment of plastic substrates may alter gene expression and prevent cellular differentiation [12,16-21], the fluid dynamics of the RWV culture system allow cells to co-localize into three-dimensional (3-D) aggregates, promoting in vivo-like exchange of growth factors and efficient cell-to-cell interactions [12-14,20,21]. This in vivo-like environment thus can promote transformed and primary cell lines to become more structurally and functionally similar to their in vivo counterparts [13,15,20-24].

In the current study we demonstrate that RWV-cultured Huh7 cells formed complex, multilayered, 3-D aggregates that exhibited up-regulation of metabolic and hepatocyte-specific transcripts as well as increased expression and re-localization of tight junction, cell adhesion, and polarity markers. Importantly, these aggregates remained highly permissive for HCV infection suggesting that hepatic polarity does not limit HCV entry in 3-D-cultured Huh7 cells. As such, RWV-cultured Huh7 cells may represent a more appropriate physiologically relevant system for further in vitro studies of HCV entry and infection dynamics.

## Methods

### Cell culture and viruses

Huh7 cells (also known as Huh7/scr cells [25,26] and Huh7-1 cells [27]) were obtained from Dr. Chisari (The Scripps Research Institute, La Jolla, CA) [2] and cultured as previously described [2]. 3-D Huh7 cultures were established using previously described techniques [13,14], with minor modifications. Briefly, 5 × 10^6^ Huh7 cells were trypisinized, incubated with 250 mg Cytodex-3 microcarrier beads (Sigma, St. Louis, MO) for 30 minutes at room temperature in a total volume of 30 ml complete DMEM. Cell-bead complexes were introduced into the RWV bioreactor (Synthecon, Inc., Houston, TX) at a ratio of 20 cells/bead, transferred to 37°C, and vessel rotation was initiated at 20 rotations per minute. Medium was replenished every 24 h and rotation speed was increased as aggregates developed to maintain cells in free-falling suspension.

Protocols for JFH-1 in vitro transcription and HCV RNA electroporation have been described elsewhere [28]. JFH-1 cell culture-propagated HCV (HCVcc) viral stocks were obtained by infection of naïve Huh7-1 cells at a multiplicity of infection (MOI) of 0.01 focus forming units (FFU)/cell, using medium collected from Huh7 cells on day 18 post transfection with in vitro transcribed pJFH-1 RNA as previously described [2].

### RNA isolation and RTqPCR

Total cellular RNA was isolated by the guanidine thiocyanate method using standard protocols [29]. One μg of RNA was used for cDNA synthesis using TaqMan reverse transcription reagents (Applied Biosystems, Foster City, CA), followed by SYBR green real-time quantitative PCR analysis (RTqPCR) using an Applied Biosystems 7300 real-time thermocycler as previously described [30]. Relative expression levels of hepatocyte-specific genes and Phase I and Phase II metabolic genes were assessed using the primers described in [30] and normalized to β-actin levels. HCV JFH-1 and GAPDH transcript levels were determined relative to a standard curve of serially diluted plasmid containing the JFH-1 cDNA or the human GAPDH gene, respectively, using primers described in [28].

### Immunofluorescence

Immunofluorescence analysis of aggregates was performed as previously described [14]. Briefly, Huh7 3-D aggregates were fixed with 4% (v/v) paraformaldehyde (Sigma, St. Louis, MO), free aldehydes quenched with 50 mM NH_4_Cl in DPBS for 10 minutes at room temperature and cells permeabilized with 0.1% Triton-X 100 (Fisher). In parallel, Huh7 2-D monolayers were seeded in 8-well chamber slides at 80% confluence and fixed 48 hours post seeding. 3-D aggregates and 2-D monolayer cells were stained with antibodies specific for scavenger receptor class B member 1 (SR-BI) (BD Biosciences, Franklin Lakes, NJ), CD81 (AbD Serotec, Raleigh, NC), CLDN1 (Abnova, Taipei, Taiwan), CD26 (Abcam, Cambridge, UK), β-Catenin (Zymed, San Francisco, CA), E-cadherin (Zymed), zona occludens 1 (ZO-1) (Zymed), Occludin (Zymed) or HCV E2 (C1 [31]) overnight at 4°C, followed by incubation with a 1:1,000 dilution of an appropriate Alexa555-conjugated secondary antibody (Molecular Probes, Carlsbad, CA) for 1 h at room temperature. Cell nuclei were stained by Hoechst dye. Bound antibodies were visualized via confocal microscopy (630×, Zeiss LSM 510, Germany). Images were analyzed using Zeiss LSM Alpha Imager Browser v4.0 software (Zeiss), and brightness and contrast were adjusted using Adobe^® ^Photoshop^® ^(San Jose, CA). Alternately, 3-D aggregates were embedded in OCT freezing medium (TissueTek, Fisher) or paraffin, sectioned and stained with Hoechst dye or Hematoxylin and Eosin (H&E), respectively.

### HCV infection kinetics

Huh7 3-D aggregates were infected with JFH-1 HCVcc at an MOI of 0.01 FFU/cell at day 1, 7 or 14 post RWV seeding by injection of the viral inoculum directly into the RWV. At indicated times post infection (p.i.), medium was harvested for titration analysis and RNA was isolated from ~0.5 ml of aggregates for reverse transcription followed by RTqPCR as described above.

### Infectivity titration assay

Culture supernatants were serially diluted 10-fold and used to infect triplicate Huh7 cultures in 96-well plates. At 24 h p.i., cultures were overlayed with complete DMEM containing 0.4% methylcellulose (Fluka BioChemika, Switzerland) to give a final concentration of 0.25% methylcellulose. Seventy-two hours p.i., cells were fixed in 4% paraformaldehyde (Sigma), and immunohistochemically stained for HCV E2 using the anti-HCV E2 antibody C1 [31]. Viral titers are expressed as FFU/ml, determined by the average E2-positive foci number detected at the highest HCV-positive dilution.

## Results

### Establishment of Huh7 3-D Aggregates

**Figure 1:**
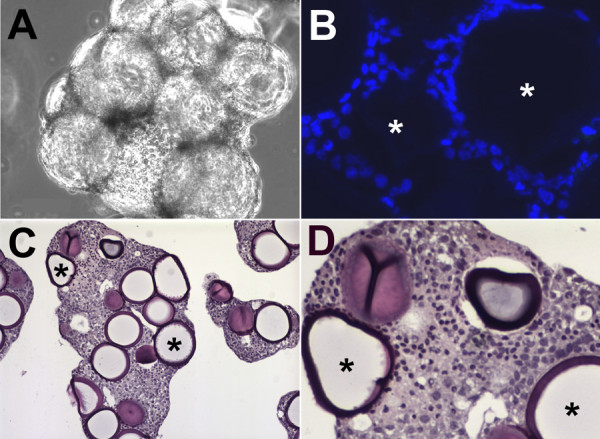
**High-fidelity 3-D Huh7 RWV aggregates**. (A) Phase contrast micrograph of Huh7 3-D aggregates cultured in the RWV for 14 days (400×). (B) Fluorescence micrograph of Hoechst-stained OCT sections of 3-D Huh7 aggregates (400×). (C-D) Light micrographs of H&E-stained paraffin sections of 3-D Huh7 aggregates [200× (C), 600× (D)]. (*) = 100 μm microcarrier bead.

To assess the utility of the RWV as a culture method for Huh7 cells, Huh7 cells were cultured on Cytodex-3 microcarrier beads in the RWV for 26 days. Morphological and cytological examination of cultures demonstrated that Huh7 cells efficiently adhered to the collagen-coated microcarrier beads and that these individual beads then assembled to form 3-D tissue-like aggregates containing ~10–20 beads per aggregate (Fig. 1A). To determine if these aggregates consisted of multilayered cells, aggregates were embedded in OCT freezing medium or paraffin, sectioned, stained with either Hoechst stain (Fig. 1B) or H&E (Fig. 1C–D) and examined by fluorescence or light microscopy, respectively. Panels C and D highlight the multilayered cellular infrastructure of the Huh7 3-D aggregates, while Hoechst's staining in Panel B illustrates similar infrastructure and confirms that the aggregates are devoid of necrotic cores.

### Gene Expression within Huh7 3-D Aggregates

**Figure 2:**
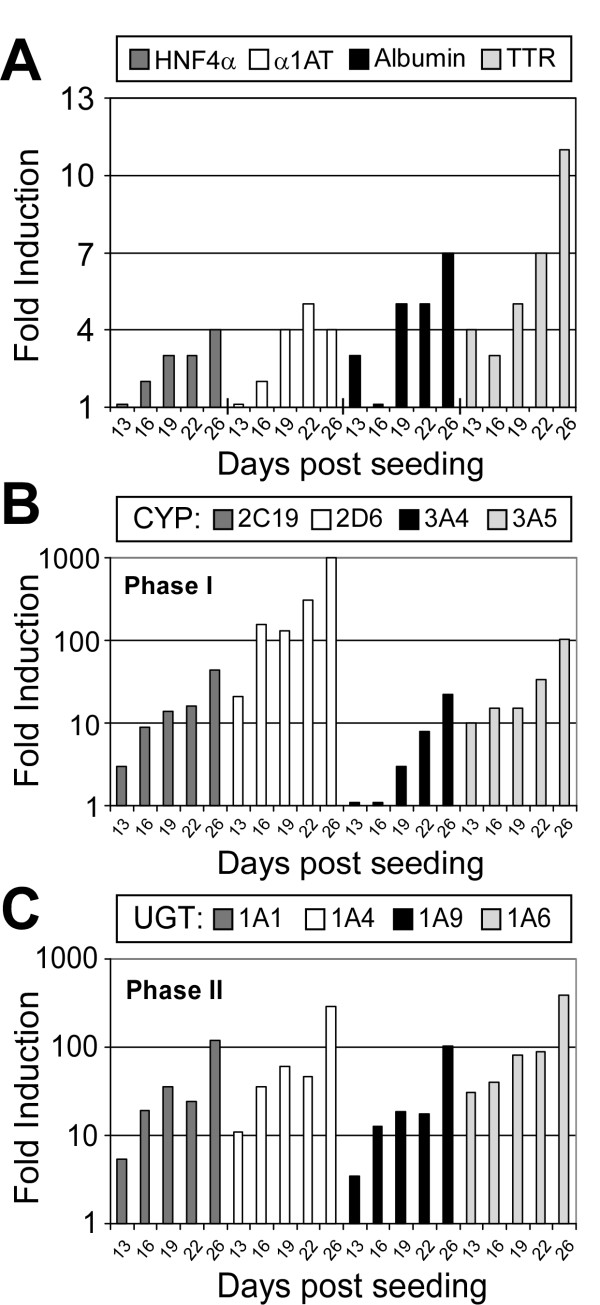
**Increased differentiation-specific gene expression in 3-D Huh7 RWV aggregates**. At indicated time points post seeding, 0.5 ml aliquots of 3-D Huh7 aggregates (~5 × 10^4 ^cells) were removed from the RWV, pelleted at 1400 RPM for 5 minutes and total RNA extracted. Expression of (A) hepatocyte-specific genes, (B) Phase I (CYP) and (C) Phase II (UGT) metabolic genes in Huh7 3-D aggregates was assessed by RTqPCR. Expression of each transcript, relative to 2-D Huh7 monolayer cultures, was determined using the  method [50], by normalizing to β-actin expression and is graphed as fold induction compared to 2-D monolayers.

One measure of hepatocyte differentiation is up-regulation of expression of transcription factors such as hepatocyte nuclear factors (HNF) [32,33], which regulate the expression of liver secretory proteins [33] such as albumin [34], alpha-1-antitrypsin (α1AT; [35]), and transthyretin (TTR; [36]). Likewise, induction of enzymes and transporters involved in Phase I and II xenobiotic metabolism [37,38], which include cytochrome P450s (CYPs) and UDP-glucuronosyltransferase (UGTs) enzymes, respectively, is another hallmark of hepatocyte differentiation. Hence, to determine whether culturing Huh7 cells in the RWV allows for cellular differentiation at the transcriptional level, expression of hepatocyte-specific genes, CYPs, and UGTs were analyzed. At indicated times post seeding, total cellular RNA was extracted from 0.5 ml of 3-D Huh7 aggregates or 2-D Huh7 monolayers grown to confluence, and relative gene expression was assessed by RTqPCR analysis. As illustrated in Fig. 2, mRNA levels for the hepatocyte-specific genes and the CYP and UGT enzymes were significantly induced in 3-D Huh7 aggregates (relative to 2-D Huh7 monolayers) and increased in a time-dependent manner while cultured in the RVW.

### Expression and Organization of Cellular Tight Junction and Polarity Markers in 3-D Huh7 Aggregates

**Figure 3:**
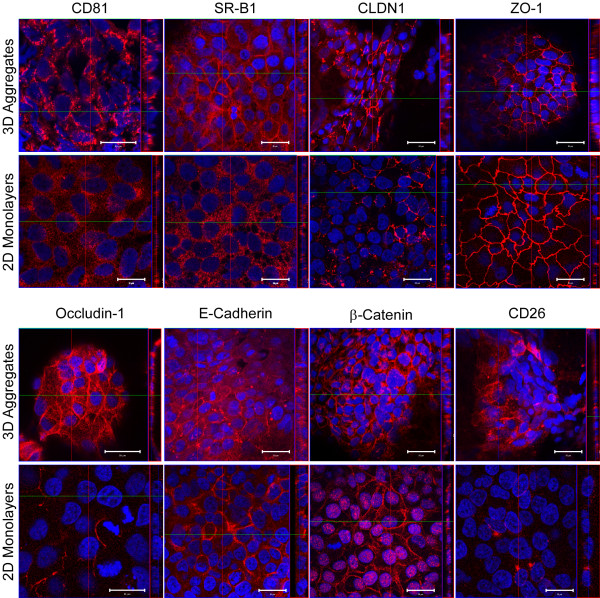
**Reorganization of HCV receptor, cell adhesion and tight junction protein localization in 3-D Huh7 aggregates**. Fourteen days post seeding, Huh7 3-D aggregates and parallel Huh7 2-D confluent monolayers were stained with antibodies specific for SR-BI, CD81, CLDN1, CD26, β-Catenin, E-cadherin, ZO-1 or Occludin and visualized via confocal microscopy (630×, Zeiss LSM 510, Germany). Small vertical panels represent x-z sections (apical = left; basal = right) of larger x-y sections, which were compiled by taking 0.5 μm steps through corresponding x-y sections. Red lines indicate the plane from which the z section was taken. The scale bar equals 20 μm.

While the effect of HCV on cell polarity and TJs (and vice-versa) cannot be accurately studied in 2-D monolayer Huh7 cultures [10], these interactions are of particular interest as TJ proteins are involved in the entry of numerous viruses [39-41] and the TJ proteins CLDN1 [7] and occludin [8,9] have recently been shown to be involved in HCV entry. Therefore, we assessed the expression and organization of the HCV receptors (CD81 and SR-B1), cell adhesion molecules (E-Cadherin and β-Catenin), cellular TJ proteins (CLDN1, ZO-1 and Occuldin-1) and the polarity marker (CD26) in 3-D Huh7 aggregates and their 2-D monolayer counterparts (Fig. 3). The expression of known HCV receptors and polarity markers were increased in 3-D Huh7 aggregates as compared 2-D Huh7 monolayers, similar to that observed by Mee et al in polarized Caco-2 cells [10]. This was not a consequence of increased mRNA levels, as normalized transcript levels for all markers examined were similar between 3-D and 2-D Huh7 cultures, as determined by RTqPCR (data not shown).

As expected, the cell adhesion molecules E-Cadherin and β-Catenin were membrane localized both in 2-D and 3-D Huh7 cultures; however, there was a profound decrease in the accumulation of nuclear β-Catenin-containing complexes in the 3-D Huh7 aggregates. Because atypical nuclear localization of β-Catenin in transformed cells has been well documented [42], the loss of this cancer-specific phenotype in the 3-D cultured Huh7 aggregates is consistent with the loss of cancer-specific markers observed in other continuous cell lines when cultured in the RWV [14,23]. Additionally, in contrast to the 2-D Huh7 monolayers, TJ markers localized to apicalateral and/or basolateral planes in the 3-D Huh7 aggregates consistent with localization patterns observed in primary hepatocytes [6,43] and normal liver tissues [44]. Finally, CD26, a cell surface ectopeptidase that localizes to the apical plane of polarized cells [45], was non-detectable in 2-D Huh7 monolayers, while, apical staining of this marker was apparent in distinct areas of 3-D Huh7 aggregates (Fig. 3). Taken together, these data demonstrate that the expression and distribution of cell adhesion and TJ proteins, including known HCV entry receptors, is enhanced and more polarized in 3-D Huh7 cultures than in 2-D monolayers.

### HCVcc Infection of Huh7 3-D Aggregates

**Figure 4:**
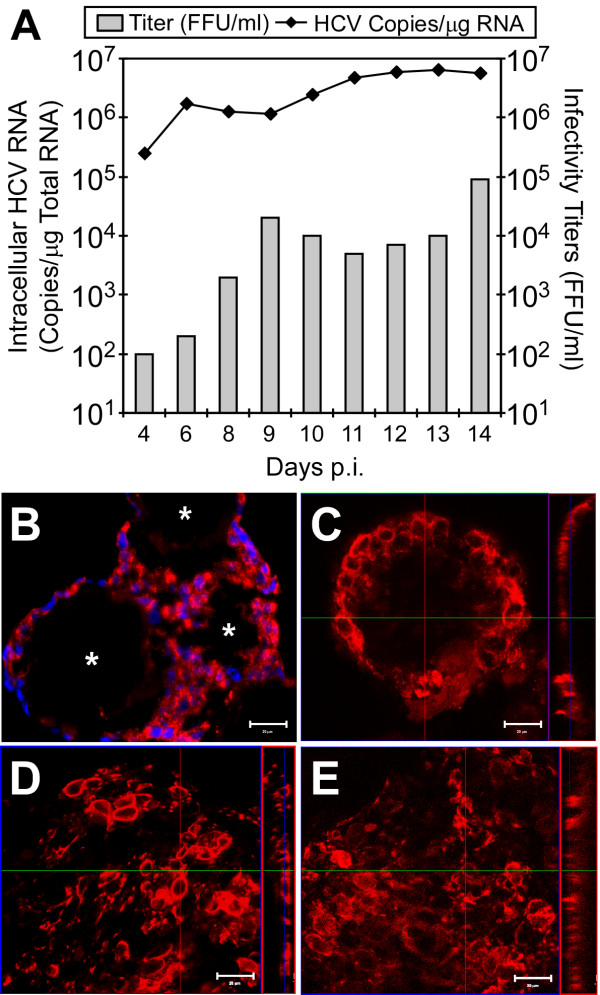
**Robust HCVcc infection in 3-D Huh7 RWV cultures**. (A) Huh7 3-D aggregates were infected with HCVcc JFH-1 at an MOI of 0.01 FFU/cell 1 day post seeding in the RWV. Culture supernatant and intracellular RNA were collected at the indicated times p.i. Normalized intracellular HCV RNA copy numbers, displayed as HCV RNA copies/μg total cellular RNA (line), were determined by RTqPCR. Infectivity titers, expressed as FFU/ml (bars), were determined by immunohistochemical analysis of 10-fold serially diluted culture supernatants on naïve Huh7 cells. (B) Indirect immunofluorescence analysis of HCV E2 expression in HCV-infected 3-D Huh7 aggregates 14 days p.i. Additional 3-D Huh7 cultures were infected on day 1 (C), 7 (D) or 14 (E) post seeding in the RWV. Aggregates were fixed 10 days p.i. and stained with a human anti-E2 antibody (C1) and anti-human-Alexa 555 secondary antibody. Images were captured via confocal microscopy (630×, Zeiss LSM 510, Germany) and Zeiss LSM Alpha Imager Browser v4.0 software (Zeiss). Image brightness and contrast were adjusted using Adobe^®^Photoshop^® ^(San Jose, CA). (*) = 100 μm microcarrier bead. Small vertical panels represent x-z sections of larger x-y sections, which were compiled by taking 0.5 μm steps through corresponding x-y sections. Red lines indicate the plane from which the z section was taken. The scale bar equals 20 μm.

Because it has been suggested that hepatocyte polarization is inversely related to the permissiveness of the cell for HCVcc infection [10,11], we sought to determine if Huh7 3-D cultures were permissive for HCVcc infection. As such, 3-D Huh7 cultures were inoculated with HCVcc JFH-1 1, 7, or 14 days post RWV-seeding and culture supernatant and cellular RNA were harvested at various time points p.i. for titration of extracellular viral titers and RTqPCR analysis of viral RNA, respectively. Fig. 4A illustrates that HCV not only infected the Huh7 3-D aggregates, but that the kinetics of HCV RNA expansion and infectious virus production increased exponentially to levels comparable to those reported using the robust 2-D Huh7 system [2,46]. To determine the percentage of cells expressing HCV proteins, indirect immunofluorescence analysis of infected 3-D Huh7 aggregates was performed. Fig. 4B shows that the majority of Huh7 cells were positive for the HCV E2 glycoprotein and that the entire aggregate was permissive for HCV infection rather than just the cells at the periphery, demonstrating that HCV can spread throughout the aggregates. Importantly, Fig. 4D and 4E illustrate that aggregates allowed to differentiate in the RWV for 7 or 14 days were as equally permissive for HCVcc infection as cells infected 1 day post RWV seeding (Fig. 4B-C), suggesting that differentiation and polarization does not negatively affect HCVcc infection in this 3-D cell culture model.

## Discussion

Here we demonstrate that Huh7 cells cultured in RWV bioreactors form multi-layered tissue-like aggregates that are phenotypically distinct from traditional Huh7 2-D monolayers (Fig. 1 and 2). Specifically, the RWV-environment promoted increases in hepatocyte-specific, as well as Phase I and II metabolic gene transcripts in 3-D Huh7 aggregates relative to Huh7 monolayers (Fig. 2). Additionally, we observed increased expression and organization of cellular HCV receptors, cell adhesion, tight junction and polarity-specific proteins, and the loss of cancer-associated nuclear localization of β-Catenin, in RWV 3-D Huh7 aggregates as compared to 2-D monolayers (Fig. 3). These data therefore suggest that the RWV environment promotes differentiation of Huh7 cells down a more hepatocyte-like route. Importantly, since these 3-D Huh7 cultures remain highly permissive for HCVcc infection, this system represents a new in vitro cell culture system for the study of HCV infection and antiviral drug studies in more polarized, xenobiotically-competent cells.

Relevant to the study of HCV, expression of the HCV receptors CD81 and SR-B1 were both diffuse and poorly organized in 2-D cultured Huh7s cells, while their expression was increased and localized to apical TJ regions and basolateral-sinusoidal surfaces in 3-D aggregates. Likewise, TJ proteins, which typically localize to the apical surface in polarized hepatocytes [43], were more concentrated at the apical surface of 3-D Huh7 aggregates as compared to monolayer cultures. Notably however, the TJ protein CLDN1, a recently identified HCV receptor [7], not only localized to TJs, but was also present at both apical and basolateral surfaces in 3-D aggregates. This localization pattern is in agreement with other studies [47] and the model proposed by Reynolds et al., describing tight-junctional (apical) and nonjunctional (basolateral) forms of CLDN1 in polarized hepatocytes [44]. As suggested by Mee et al, it may be that these non-junctional pools of CLDN1 have a direct role in HCV entry [11]. Interestingly, Battle et al., have demonstrated a correlation between HNF4α and cell adhesion and TJ molecules expression and organization [48]. Whether this is also the case in the 3-D Huh7 aggregates, which have increased HNF4α expression (Fig. 2A) remains to be determined. Nonetheless, the ability of 3-D cultured Huh7 cells to better organize cell adhesion and TJ proteins is a phenotype consistent with other RWV-cultured cell types [14,21,23]. As such, RWV-cultured Huh7 cells provide an appropriate model for investigating HCV entry, particularly the interaction, organization, and stoichiometry of HCV receptors and TJ proteins. Additional analyses to determine the extent of differentiation and polarization of 3-D Huh7 aggregates is still warranted and a focus of ongoing studies.

To date, attempts to study HCV in polarized cells have been limited to colorectal adenocarcinoma Caco-2 cells [10] or HepG2 cells [11], neither of which support robust HCVcc infection. Although an inverse relationship between cell polarization and HCV entry into polarized Caco-2 [10] and HepG2 [11] cells has been observed no such phenotype was observed in 3-D Huh7 aggregates. Specifically, 3-D Huh7 aggregates, infected at various stages of differentiation (e.g. day 1, 7 or 14 post seeding), were equally permissive for HCVcc infection (Fig. 4B–E). Furthermore, 3-D aggregates treated with PMA, a known disruptor of TJ formation [49], were no more permissive for HCV infection as compared to untreated parallel aggregates (data not shown), suggesting that the TJ barriers formed in 3-D Huh7 aggregates are not inhibitory for HCVcc infection.

## Conclusion

Growing evidence suggests interplay between TJ protein expression, localization and function and HCV infection. Although, the current HCV infectious 2-D Huh7 cell culture system does not amend itself well to elucidating these dynamic relationships, the highly HCV-permissive 3-D Huh7 cell culture system described herein more closely mimics the differentiated and polarized state of hepatocytes. As such the RWV 3-D Huh7 cell culture system should prove useful for understanding the dynamic relationship between HCV and TJ protein expression as well as elucidating how HCV interacts with and disrupts key aspects of hepatocyte physiology.

## Abbreviations

HCV: hepatitis C virus; JFH-1: Japanese Fulminant Hepatitis; RWV: rotating wall vessel; 3-D: three dimensional; 2-D: two-dimensional; HCVcc: hepatitis C virus cell-cultured produced; MOI: multiplicity of infection; FFU: focus forming units; RTqPCR: real-time quantitative PCR; SR-B1: scavenger receptor class B member 1; CLDN1: claudin-1; ZO-1: zona occludens 1; H&E: hematoxylin and eosin; p.i.: post infection; HNF: hepatocyte nuclear factors; α1AT: alpha-1-antitrypisn; TTR: transthyretin; CYP: cytochrome P450s; UGT: UDP-glucuronosyltransferase; TJ: tight junction.

## Competing interests

The authors declare that they have no competing interests.

## Authors' contributions

BS and VT participated in the design of the study, performed the experiments and drafted the manuscript. SLU designed the study and participated in drafting the manuscript. All authors read and approved the final manuscript.
